# Kinetin Ameliorates Cisplatin-Induced Hepatotoxicity and Lymphotoxicity via Attenuating Oxidative Damage, Cell Apoptosis and Inflammation in Rats

**DOI:** 10.3390/biomedicines10071620

**Published:** 2022-07-06

**Authors:** Moustafa Fathy, Mostafa A. Darwish, Al-Shaimaa M. Abdelhamid, Gehad M. Alrashedy, Othman Ali Othman, Muhammad Naseem, Thomas Dandekar, Eman M. Othman

**Affiliations:** 1 Department of Biochemistry, Faculty of Pharmacy, Minia University, Minia 61519, Egypt; mostafa_fathe@minia.edu.eg; 2 Department of Pharmacology and Toxicology, Faculty of Pharmacy, Nahda University, Beni-Suef 62521, Egypt; mostafa.darwish@nub.edu.eg; 3 Department of Chemistry, Biochemistry Division, Faculty of Science, Minia University, Minia 61519, Egypt; shimaamostfa223@gmail.com (A.-S.M.A.); biochemist.gehad.mahmoud@gmail.com (G.M.A.); osman.mouftah@mu.edu.eg (O.A.O.); 4 Department of Life and Environmental Sciences, College of Natural and Health Sciences, Zayed University, Abu Dhabi 144534, United Arab Emirates; muhammad.naseem@zu.ac.ae; 5 Department of Bioinformatics, Biocenter, University of Wuerzburg, Am Hubland, 97074 Wuerzburg, Germany

**Keywords:** cisplatin, hepatotoxicity, lymphotoxicity, oxidative stress, AKT, CD95, caspase-3

## Abstract

Though several previous studies reported the in vitro and in vivo antioxidant effect of kinetin (Kn), details on its action in cisplatin-induced toxicity are still scarce. In this study we evaluated, for the first time, the effects of kinetin in cisplatin (cp)- induced liver and lymphocyte toxicity in rats. Wistar male albino rats were divided into nine groups: (i) the control (C), (ii) groups 2,3 and 4, which received 0.25, 0.5 and 1 mg/kg kinetin for 10 days; (iii) the cisplatin (cp) group, which received a single intraperitoneal injection of CP (7.0 mg/kg); and (iv) groups 6, 7, 8 and 9, which received, for 10 days, 0.25, 0.5 and 1 mg/kg kinetin or 200 mg/kg vitamin C, respectively, and Cp on the fourth day. CP-injected rats showed a significant impairment in biochemical, oxidative stress and inflammatory parameters in hepatic tissue and lymphocytes. PCR showed a profound increase in caspase-3, and a significant decline in AKT gene expression. Intriguingly, Kn treatment restored the biochemical, redox status and inflammatory parameters. Hepatic AKT and caspase-3 expression as well as CD95 levels in lymphocytes were also restored. In conclusion, Kn mitigated oxidative imbalance, inflammation and apoptosis in CP-induced liver and lymphocyte toxicity; therefore, it can be considered as a promising therapy.

## 1. Introduction

Cisplatin (CP) is one of the most widely used and effective anti-neoplastic drugs. It is used to treat a wide variety of cancers, including those that affect the testicles, ovaries, cervical region, bladder, head and neck, as well as the lung [1]. CP induces many toxicities, including nephrotoxicity, hepatotoxicity, cardiotoxicity, neurotoxicity and ototoxicity, which restrict its clinical usage [2,3,4]. A large number of experimental research papers have provided evidence of the prevalent toxic side effects associated with CP, most notably nephrotoxicity [5]. Additionally, it has been shown that hepatotoxicity is a prominent dose-limiting adverse effect of CP-based chemotherapy [6,7]. Furthermore, accumulating evidence points to cisplatin’s ability to induce genotoxicity/DNA damage in bone marrow and human lymphocytes [8]. The production of reactive oxygen species (ROS), including superoxide anions and hydroxyl radicals, which leads to an increase in lipid peroxidation and cell damage, is what drives the toxicity that is induced by CP. Likewise, apoptosis and cell arrest are events resulting from an excess of reactive oxygen species in cancer cells as well as non-target normal cells [9,10]. Although the increased oxidative stress and inflammation are the main mechanisms involved in toxicity induced by CP, other molecular pathways have yet to be revealed.

Recently, considerable focus has been placed on the protective function that naturally occurring compounds with antioxidants and anti-inflammatory characteristics provide, as well as the mechanism by which they exert their effects. Cytokinins (CKs) are natural products that have lately come to the forefront of attention. These natural compounds have a variety of therapeutic and pharmacological effects [11]. Researchers were prompted to investigate the process of CK production at the nucleic acid level due to the prevalence of CKs in DNA and cell extracts [12]. The small-molecule adenosine N6-furfuryladenine (N6FFA: kinetin) is commonly used by the plant community as a low-priced proxy for the natural cytokinins (CKs) in plant tissue culture and other experiments relevant to plant growth and development [11,13].

A long time ago, Kn was thought to be an artificial product of DNA rearrangement [14], but it was then discovered in extracts from Casuarina equisetifolia root nodules infected with the bacteria Frankia in 1996, which was the first evidence of its natural occurrence [15]. In addition, and in the same year, Barciszewski et al., discovered kinetin in plant cell extracts (0.1 ng/g dried material), DNA extracted from human fibroblasts cultivated in vitro and commercially available calf thymus DNA [16].

Kinetin was found in the endosperm liquid of fresh young coconut fruits, plant cell extracts, human urine and various other biological extracts [11].

Furthermore, the free radical scavenging activity of kn was investigated in vitro in inactivated platelets, and the findings show that Kn inhibited the generation of hydroxyl radicals in a dose-dependent manner, as well as exerting antithrombotic action in three in vivo models [17]. Besides its antioxidant activity, Othman et al., (2021) have provided evidence of the anti-inflammatory action of kinetin, suggesting that it can be used as a potential therapeutic agent against cellular inflammatory responses and oxidative stress arising from a wide range of pathophysiological conditions [18]. The fact that kn has been shown to be useful in guarding against oxidative damage and inflammation demonstrates its potential as an adjuvant in the treatment and prevention of a variety of clinical conditions. However, up to this point in time, no research has been carried out to determine the protective impact of Kn against the hepatotoxicity and lymphocyte toxicity that are caused by CP. Therefore, the purpose of this study was to pull the curtain to unveil the possible protective effects of Knin CP-induced liver and lymphocyte toxicity that was displayed at different doses and to investigate the mechanisms that explain these possible effects, regarding its possible influence on oxidative stress and inflammation.

## 2. Materials and Methods

### 2.1. Kits, Chemicals and Antibodies

Kinetin (Kn) was obtained from Sigma-Aldrich (Dorset, Germany). Cisplatin was purchased from Mylan SAS pharmaceutical company (Saint-Priest, France). Kits for reduced glutathione (GSH) (CAT No. GR2511), Glutathione peroxidase (GPx) (CAT No. GP 2524), Malondialdehyde (MDA) (CAT No. MD2529) and Superoxide dismutase (SOD) were purchased from Biodiagnostic (Cairo, Egypt). Tumor Necrosis Factor α (TNF- α), AKT and Caspase-3 polyclonal antibodies were purchased from Santa Cruz Biotechnology (Dallas, TX, USA). Rat ELISA Kits of TNF- α, CRP and Interleukin 6 (IL-6) were obtained from Elabscience biotechnology (Houston, TX, USA) while rat FAS/CD95 (Factor-Related Apoptosis) ELISA Kit was purchased from Assay Genie, Ireland (CAT No.: RTFI00768). Histopaque -1077 was purchased from Sigma-Aldrich (Dorset, Germany (CAT No. 10771). Rat ELISA Kit for pAKT/AKT was purchased from Bioassay Technology Laboratory (Shanghai, China).

### 2.2. Study Design

All study protocols involving animals were approved by the Institutional Animal Care and Use Committee at Minia University, Egypt (Permit Number: 54/2019). Adult male Wistar rats (Animal Care Unit, Faculty of Agriculture, Minia University, weighing 140–160 g) were fed standard laboratory chow (El-Nasr Company, Cairo, Egypt) and water ad libitum. Rats were accommodated at 22 °C ± 2 °C with humidity of 50% ± 10% with 12 h dark–light cycle. After a 2-week acclimatization period, rats were randomly divided into 9 groups (6 animals each). Control group received only vehicle for 10 days, (0.25 Kn group) received a dose of 0.25 mg/kg Kn, (0.5 Kn group) received a dose of 0.5 mg/kg Kn and (1.0 Kn group) received a dose of 1.0 mg/kg kinetin. Kn at all different doses was given in a single daily dose for 10 days, i.p. Kn was freshly prepared in saline as a vehicle (0.9% M/V NaCl). (CP group): a single intraperitoneal dosage of 7.0 mg/kg cisplatin was administered on the 4th day of the experiment. (CP + 0.25 Kn group) rats received i.p. 0.25 mg/kgKn once daily for 10 days along with a single i.p. dose of 7.0 mg/kg cisplatin on the 4th day of the experiment, (CP + 0.5 Kn group) rats were given 0.5 mg/kg Kn once daily, i.p., for 10 days concomitant with a single i.p. injection of 7.0 mg/kg cisplatin on the 4th day of the experiment, (CP + 1.0 Kn group) animals received 1.0 mg/kg Kn, i.p., once daily for 10 days concomitant with a single i.p. injection of 7.0 mg/kg cisplatin on the 4th day of the experiment, and (CP + Vit C group): rats were given 200 mg/kg vitamin C once daily for 10 days orally in addition to a single i.p. injection of 7.0 mg/kg cisplatin on the 4th day of the experiment. The dose and experimental design of Kn and CP administration were guided by our previous work [19].

### 2.3. Serum and Tissue Sample Collection

After ten days (the period of the experiment), rats were anesthetized using diethyl ether and before sacrificing; blood samples were collected by small capillary tubes from the retro-orbital plexus, and serum samples were prepared by centrifugation at 3000 rpm for 10 min in a refrigerated centrifuge and stored at −80 °C.

After euthanization by cervical dislocation, liver tissue samples were collected for the histopathological, biochemical and PCR analyses.

For the histopathological analysis, parts of liver tissues were fixed by 10% formalin, while for the biochemical and PCR analyses, other parts were frozen with using liquid nitrogen and stored at −80 °C.

### 2.4. Lymphocyte Isolation

Lymphocyte isolation was performed as described previously with some modifications [20]. In brief, 7 mL of blood from several groups of rats was applied as a layer atop Histopaque-1077 (1:1). The layers were separated by centrifugation (400× *g*) at room temperature for 30 min, after which mononuclear cells were recovered from the plasma–Histopaque interface. They were resuspended in lymphocyte medium after being washed twice with lymphocyte medium (250× *g*; 1300 rpm; room temperature for 10 min). Lymphocytes were incubated for 24 h with 100 nM Kn and 30 min with 0.6 μM NQO after that, and various parameters were assessed.

### 2.5. Biochemical Assays

For the measurement of the levels of ALT, AST, ALP, Alb and bilirubin in serum samples, commercially available colorimetric assay kits (Sigma-Aldrich) were used, and the standard procedures were applied.

### 2.6. Lipid Peroxidation and Antioxidant Status Evaluation

The levels of MDA and GSH as well as GPx and SOD activities were assessed in the liver homogenates and lymphocytes using commercially available kits (Biodiagnostic, Cairo, Egypt) following the manufacturer’s instructions.

### 2.7. Histopathological Examination

For the histopathological examinations, the previously fixed liver tissues in 10% formalin were removed, dehydrated in ethanol and prepared as blocks in paraffin wax. Liver sections 4–5 µm-thick were prepared on clean, dry glass slides, deparaffinized and stained using hematoxylin and eosin [21]. The sections were then examined and visualized at 200× magnification using a light microscope (Leika DMRBE, Wetzlar, Germany) to detect pathological changes.

### 2.8. Quantitative Real-Time Polymerase Chain Reaction (qRT-PCR)

RiboZol reagent was used for isolating total RNA from the hepatic tissue through a series of steps. The RNA was quantified using a NanoDrop spectrophotometer, and the mentioned amount of cDNA was produced using the manufacture protocol of GoTaq^®^ One-Step RT-qPCR System (Madison, WI, USA). Table 1 depicts the primer sequences. The results were normalized to Glyceraldehyde 3-phosphate dehydrogenase (GAPDH), which was used as internal control. The experiments were performed in triplicate.

### 2.9. Western Blot Analyses

Western blot was performed in accordance with the method previously described [22], and ready Prep™ buffer for protein extraction (Bio-Rad Inc., catalog #163-2086) was used, including lysing of the isolated lymphocytes from blood of all experimental animal groups. Lysates were centrifuged, and protein contents were determined using a Bradford protein assay kit. Following that, 20 µg of protein from each sample was combined with Laemmli loading buffer and separated on 10% sodium dodecyl sulfate–polyacrylamide gels. Blotting of separated protein contents was then performed on nitrocellulose membrane (Millipore, Burlington, MA, USA). Following blocking with 5% skim milk, the membrane was incubated with primary antibodies against TNF-α and β-actin, followed by secondary antibodies (HRP-conjugated goat IgG). The proteins were visualized using the enhanced chemiluminescent kit. The membrane was re-probed with β-actin to demonstrate equivalent loading of the individual sample proteins. Densitometry was performed by Image J 7.0 for semi-quantification of protein bands relative to β-actin, and the results are displayed as a bar chart.

### 2.10. Statistical Analysis

Data were expressed as mean ± SEM and statistically analyzed using one-way analysis of variance (ANOVA) for multiple-group comparisons followed by Tukey–Kramer post hoc test using GraphPad Prism, version 6 (San Diego, CA, USA), with differences considered significant at *p* < 0.01.

## 3. Results

### 3.1. Effect on Hepatic Biochemical Parameters

**Figure 1:**
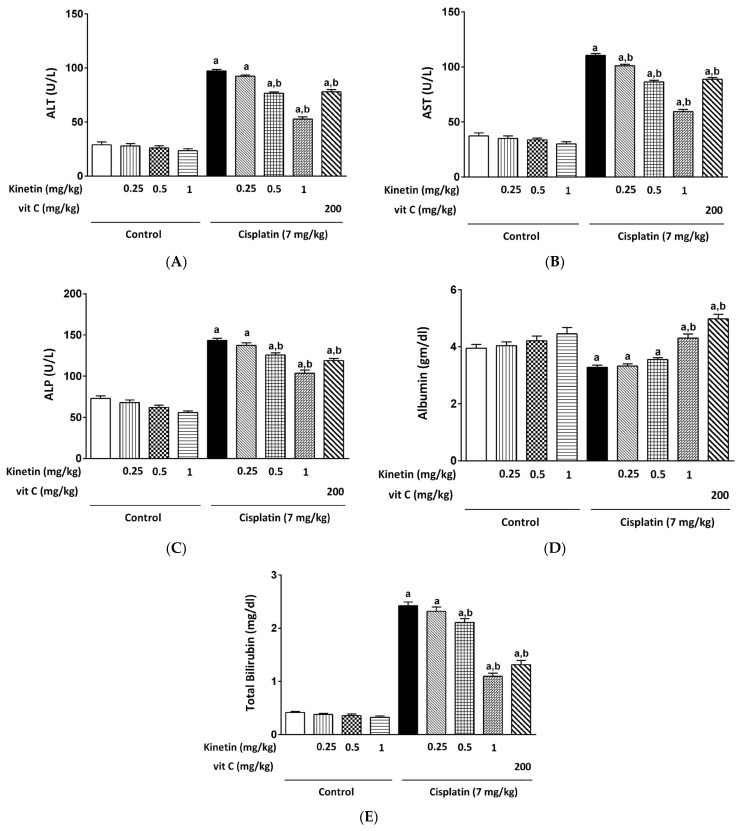
Effect of kinetin on different biochemical parameters. Effect of different doses of Kn and vitamin C on serum (**A**) ALT, (**B**) AST, (**C**) ALP, (**D**) albumin and (**E**) total bilirubin. a Significantly different from control group, b significantly different from cisplatin group according to one-way ANOVA followed by Tukey–Kramer multiple comparisons test at *p* < 0.01, (*n* = 6). Tests were performed as described in Materials and Methods. ALT: alanine transferase, AST: aspartate transferase, ALP: alkaline phosphatase, Vit C: vitamin C.

Kinetin (Kn) was given at different doses (0.25, 0.5 and 1 mg/kg) to obtain insights into the impact of the different doses of Kn treatment on CP-induced hepatotoxicity. All doses administered to Kn-treated rats displayed non-significant changes in serum ALT, AST, ALP, albumin and total bilirubin compared to the normal control group. CP significantly increased levels of ALT, AST, ALP and total bilirubin while markedly decreasing serum bilirubin as compared to the normal control group. Remarkably, the lower dose of Kn (0.25 mg/kg) did not mitigate the level of the abovementioned function parameters except for in the case of AST levels as compared to the CP group (Figure 1B). However, higher doses such as 0.5 and 1 mg/kg caused a significant attenuation in the serum level of the aforementioned parameters in CP-treated rats (Figure 1A–E). The vitamin C group, which serves as a standard antioxidant, showed a significant improvement in all these functional markers.

### 3.2. Effect on Hepatic Redox Status

**Figure 2:**
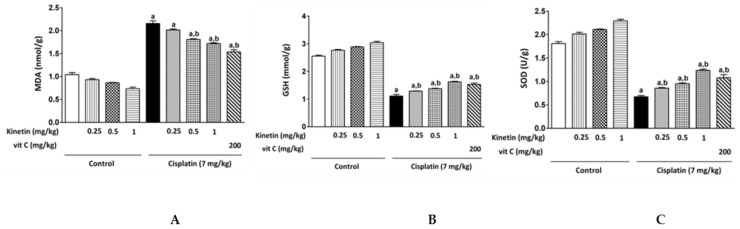
Effect Effect of kinetin oon hepatic redox status. Effect of different doses of kinetin and vitamin C on hepatic (**A**) MDA, (**B**) GSH and (**C**) SOD. a Significantly different from control group, b significantly different from cisplatin group according to one-way ANOVA followed by Tukey–Kramer multiple comparisons test at *p* < 0.01, (*n* = 6). Tests were performed as described in Research Design and Methods. MDA: malondialdehyde, GSH: glutathione, SOD: superoxide dismutase, Vit C: vitamin C.

Cisplatin administration induced oxidative stress in rat liver. It significantly elevated MDA content while remarkably decreasing GSH content and SOD activity when compared to the control rats. On the contrary, Kn, at all doses except for the lowest dose (0.25 mg/kg), successfully ameliorated the above oxidative stress parameters induced by CP in rat liver (Figure 2).

### 3.3. Effect on TNF-α Level in Liver Homogenates

**Figure 3:**
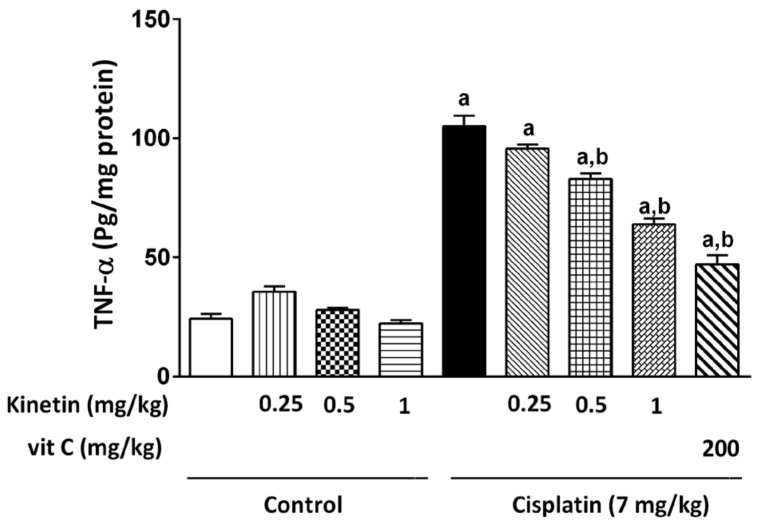
Effect Effect of kinetin on hepatic TNF-α level. Effect of different doses of kinetin and vitamin C on hepatic TNF-α level. a Significantly different from control group, b significantly different from cisplatin group according to one-way ANOVA followed by Tukey–Kramer multiple comparisons test at *p* < 0.01, (*n* = 6). Tests were performed as described in Research Design and Methods. TNF-α: Tumor necrosis factor- α, Vit C: vitamin C.

Hepatic TNF-α concentration was examined as an inflammatory marker that showed a significant increase in the CP group when compared to the control group. Prior administration of Kn at all doses in addition to Vit C significantly reduced the hepatic TNF-α concentration compared with the CP group (Figure 3).

### 3.4. Effect on Redox Status in Lymphocytes

**Figure 4:**
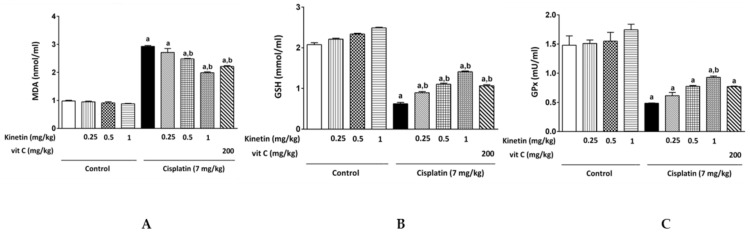
Effect Effect of kinetin on redox status in lymphocytes. Effect of different doses of kinetin and vitamin C on (**A**) MDA, (**B**) GSH and (**C**) GPx in lymphocytes. a Significantly different from control group, b significantly different from cisplatin group according to one-way ANOVA followed by Tukey–Kramer multiple comparisons test at *p* < 0.01, (*n* = 6). Tests were performed as described in Research Design and Methods. MDA: malondialdehyde, GSH: glutathione, GPx: Glutathione peroxidase, Vit C: vitamin C.

Oxidative stress markers, such as lipid peroxide content, MDA level, GSH concentration and GPx activity, were assessed for oxidative stress status evaluation in lymphocytes. Injection with CP caused a remarkable rise in the MDA level as well as a significant decline in GSH concentration and GPx activity compared with the control (Figure 4A–C). Concomitant treatment of Kn at different doses (0.25, 0.5 and 1.0 mg/kg) or Vit C significantly restored the GSH level compared to the CP-treated group (Figure 4B). Interestingly, the highest two doses of Kn in addition to Vit C significantly mitigated oxidative stress in the case of MDA levels in comparison with the CP-treated group, whereas a 0.25 mg/kg Kn dose did not prevent the toxicity induced by CP (Figure 4A). Notably, concomitant administration of 0.25 and 0.5 mg/kg Kn doses or Vit C did not significantly restore the GPx activity when compared to the CP group, while in contrast, only the 1 mg/kg Kn significantly attenuated the remarkable reduction in GPx activity induced in CP-treated animals (Figure 4C).

### 3.5. Effect on Inflammatory Status in Lymphocytes

**Figure 5:**
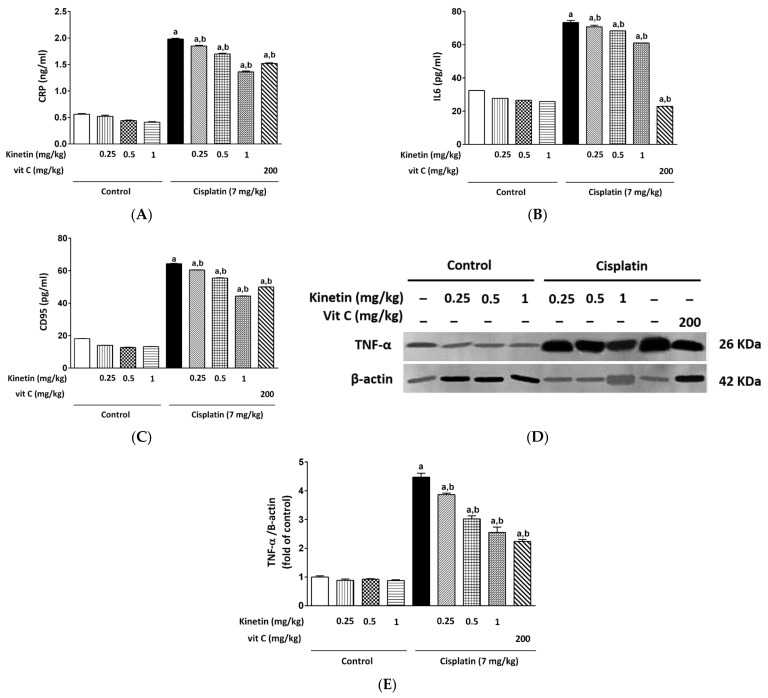
Effect Effect of kinetin on inflammatory status in isolated lymphocytes. Effect of different doses of kinetin and vitamin C on (**A**) CRP, (**B**) IL6 and (**C**) CD95. (**D**) Western blot analysis showing effect of different doses of kinetin and vitamin C using TNF-α Ab together with β-actin Ab as an internal control showing strong activation of TNF-α in CP-treated group (*n* = 6). (**E**) Quantitative analysis (fold changes) of Western blots. a Significantly different from control group, b significantly different from cisplatin group according to one-way ANOVA followed by Tukey–Kramer multiple comparisons test at *p* < 0.01, (*n* = 6). Tests were performed as described in Research Design and Methods. CRP: C-reactive protein, IL6: interleukin 6, CD95: cluster of differentiation, Vit C: vitamin C.

Cisplatin induces inflammation, which is a crucial pathway in its toxicity. Therefore, we tested levels of CRP, IL6, CD95 and TNF-α protein expression as inflammatory biomarkers. A significant elevation in CRP, IL6, CD95 and TNF-α protein levels was observed in the CP-treated group compared to the control group (Figure 5). Intriguingly, Kn administration at doses of 0.25, 0.5 and 1.0 mg/kg or Vit C along with CP caused a significant decrease in CRP, IL6 and CD95 levels as well as downregulation of TNF-α protein expression (Figure 5D,E) as compared to the CP group.

### 3.6. Effect on Hepatic Histological Features

**Figure 6:**
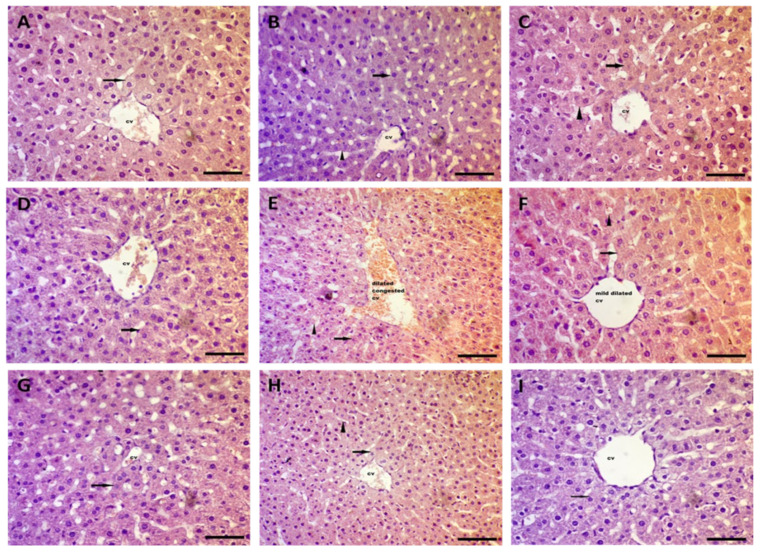
Photomicrographs of rats’ livers (H & E × 200). (**A**) Normal control rat sections showed normal architecture with normal central veins **cv** in the center of hepatic lobule with hepatocytes peripherally arranged, as shown by arrows. (**B**–**D**) Normal rats injected with Kn at different doses (0.25, 0.5 and 1.0 mg/kg, respectively) displayed normal architecture with mild dilated central veins in the center of hepatic lobule with hepatocytes peripherally arranged, as shown by the arrows, and separated by sinusoids (arrow head). (**E**) Hepatic sections of CP (7.0 mg/kg)-treated group showed marked dilation and congestion of the central veins in the center of hepatic lobule with hepatocyte degeneration near central vein and were peripherally arranged with a vacuolated to clear cytoplasm, and separated by dilated and congested sinusoids (arrow head). (**F**,**G**) Combination of cisplatin with Kn (0.25 and 0.5 mg/kg) restored the normal architecture of hepatocytes with mild congestion in central veins in the center of hepatic lobule with hepatocytes peripherally arranged (arrow) and separated by sinusoids (arrow head). (**H**) The highest Kn dose (1.0 mg/kg) restored the normal architecture of hepatocytes. (**I**) Combination of cisplatin with 200 mg/kg vitamin C restored the normal architecture of hepatocytes with mild congestion in central veins in the center of hepatic lobule with hepatocytes peripherally arranged (arrow) and separated by sinusoids (arrow head), (H & E × 200).

In Figure 6, normal rats injected with Kn at all doses displayed normal architecture with mild dilated central veins cv in the center of the hepatic lobule with hepatocytes peripherally arranged, as shown by the arrow, and separated by sinusoids (arrow head) compared to the normal control group. On the other hand, hepatic sections of the CP-treated group showed marked dilation and congestion of the central veins cv in the center of the hepatic lobule with hepatocyte degeneration near the central vein, were peripherally arranged, with a vacuolated to clear cytoplasm, and separated by dilated and congested sinusoids (arrow head). Concomitant administration of Kn in 0.25 and 0.5 mg/kg doses and Vit C treatment for CP-injected rats restored the normal architecture of hepatocytes with mild congestion in central veins cv in the center of the hepatic lobule with hepatocytes peripherally arranged (arrow) and separated by sinusoids (arrow head). Intriguingly, the highest Kn dose (1.0 mg/kg) restored the normal architecture of hepatocytes in hepatic histopathological sections when compared to the CP-treated group.

### 3.7. Effect on Hepatic AKT, Caspase-3 Gene Expression and pAKT/AKT Ratio

**Figure 7:**
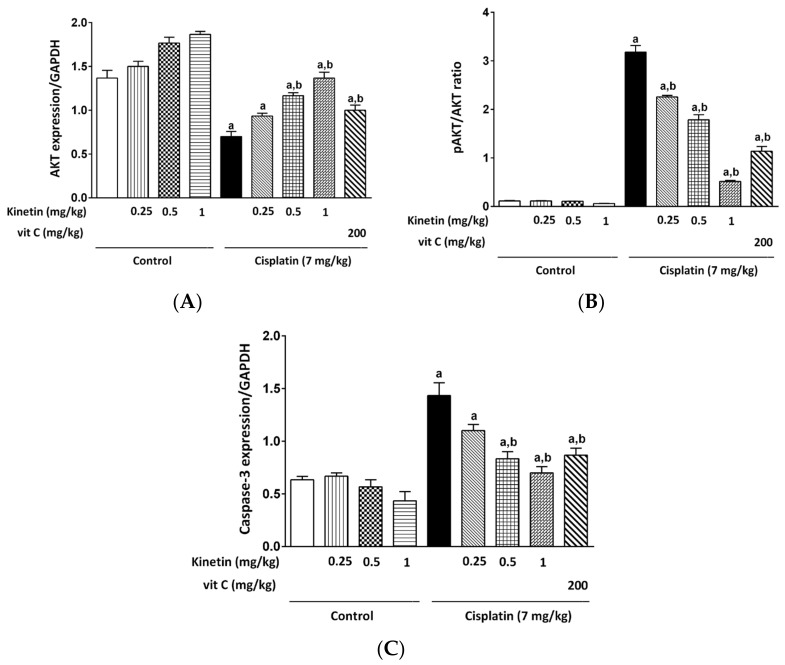
Effect Effect of kinetin on hepatic AKT, Caspase-3 gene expression and pAKT/AKT ratio. Effect of different doses of kinetin and vitamin C on (**A**) AKT gene expression, (**B**) PAKT/AKT ratio and (**C**) Caspase-3 gene expression. a Significantly different from control group, b significantly different from cisplatin group according to one-way ANOVA followed by Tukey–Kramer multiple comparisons test at *p* < 0.01, (*n* = 6). Vit C: vitamin C.

A significant decrease in AKT expression and a marked increase in caspase-3 expression in addition to the pAKT/AKT ratio, as apoptotic markers, were found in the liver of CP-injected animals in comparison with normal rats, as shown by PCR and ELISA analyses, respectively. Treatment with Kn at all doses as well as Vit C prior to CP injection caused a marked rise in the expression of the AKT gene, whereas a remarkable decline was observed in caspase-3 gene expression as well as the pAKT/AKT ratio when compared with the CP-injected rats. No marked changes in AKT, caspase-3 gene expressions or the pAKT/AKT ratio were found in the Kn-injected groups at any of the doses in comparison with normal animals (Figure 7A–C).

## 4. Discussion

In 2016, our research team published a study in which we demonstrated that Kn had both an antioxidant and an antigenotoxic impact on a variety of mammalian cell lines representing various organs. In addition, we emphasized that CKs might have multiple activities depending on the dose that was administered [18], and another study confirmed our findings by demonstrating that antioxidant activity is a mechanism for the antigenotoxic impact of Kn [23]. Moreover, Kn was demonstrated to have the highest antioxidant activity up to a concentration of 1 µM among four CKs chosen by Bizzalori [24]. Our first study was followed by another study which discussed the safety of the systemic use of Kn [18]. Accordingly, in the present work, we investigated the protective effect of Kn at different doses against CP-induced toxicity in the liver as well as isolated lymphocytes. To the best of our knowledge, this is the first report exploring the possible molecular protective mechanisms of Kn concerning its antioxidant, anti-inflammatory and anti-apoptotic properties against CP-induced hepatotoxicity and lymphotoxicity, which have not been previously studied.

Cisplatin is one of the most potent cytotoxic anticancer medicines; nonetheless, hepatotoxicity and lymphotoxicity are two of its significant adverse effects, which represent a major hurdle limiting its usage. Furthermore, they have been linked to the excessive generation of ROS and cell and DNA damage [25]. Furthermore, oxidative stress causes inflammation and the generation of cytokines such as TNF-a and IL-6.

In this context, our study demonstrated that CP treatment resulted in a severe array of events of hepatotoxicity as evidenced by significant elevation of ALT, AST, ALP and total bilirubin while a marked hypoalbuminemia was accompanied by distorted architecture and severe degenerative changes in the hepatocytes. The potential of CP to increase serum ALT, AST and ALP activity is thought to be a secondary event after CP-induced liver injury and the subsequent leakage of these enzymes from hepatocytes [26]. Conjointly, elevated bilirubin levels may be caused by either decreased hepatic bilirubin uptake or a decreased rate of bilirubin conjugation in the liver [27]. In agreement of our results, Okoko and Ndoni (2018) reported an elevation in the serum ALT, AST and ALP activities in CP-induced hepatotoxicity [28]. Notably, the remarkable hypoalbuminemia reported with CP injection in our study was the same as previously reported by Neamatallah et al., (2018) [2]. Interestingly, concomitant treatment with Kn, however, restored the normal architecture of hepatocytes as well as the aforementioned functional parameters of the liver.

Excess ROS generation in CP-treated rats resulted in an imbalance in oxidant–antioxidant levels, which lowered scavenging capability toward ROS and promoted oxidative stress. This was emphasized in our results by the elevation in both hepatic and lymphocyte MDA content, along with a diminution of the enzymatic antioxidants, including hepatic SOD as well as GPx, in lymphocytes. Moreover, the depletion of GSH levels in CP-induced rats made the hepatic tissue as well as lymphocytes more susceptible to oxidative stress. This is similar to the results obtained by Bentli et al., (2013) [29], Omar et al., (2016a) [7]; Omar et al., (2016b) [30] and Yadav et al., (2019) [31]. Importantly, in the current study, administration of Kn along with CP showed protection against oxidative stress compared to the CP group. The potential protection of Kn against lipid peroxidation and depletion of antioxidant enzymes may reveal a plausible mechanism of kinetin’s antioxidative action. Olsen et al., (1999) showed how Kn protects DNA from oxidative damage by preventing ROS generation and scavenging them before their reaction with the DNA [32].

Our findings are consistent with prior research that found Kn to be effective in lowering MDA levels and increasing antioxidant enzymes in spermatozoa and other mammalian cells [28,29]. Additionally, in another study using mouse models, Kn had the ability to enhance the antioxidant system of the cell by increasing the activities of SOD, GSH-px and heme oxygenase 1 (HO-1), where oxidative damage was induced by aluminum chloride and D-galactose [33]. Interestingly, we noticed that Kn had intrinsic antioxidant activity when administered alone and acted as antioxidant upon treatment in combination with CP in a dose-dependent manner. In this regard, the highest concentration of Kn (1.0 mg/kg) showed the highest antioxidant potential among all the doses, and this effect was comparable to or even greater than the effect of the potent antioxidant vitamin C. Accordingly, we observed that CP-treated rats, which were administered the dose of 1.0 mg/kg kinetin, showed the highest recovery of the functional markers when compared with the other doses of Kn. Similarly, Abdel-latif et al., (2022) showed that Kn provided protection against oxidative-stress-induced testicular damage in CP-treated rats, again in a dose-dependent manner [19].

Different studies reported that CP-induced oxidative stress enhances the process of inflammation and TNF-α production, which leads to the activation of a large network of pro-inflammatory cytokines such as IL-1β and IL-6 [2,34,35].

During hepatotoxic damage, pro-inflammatory cytokines such as TNF-, IL-1 β and IL-6 are released into the circulation by the liver [36]. In the current investigation, hepatic oxidative stress induced by a single dosage of CP (7.0 mg/kg) was predicted to promote inflammation as assessed by substantial TNF-α upregulation in both hepatic tissue and lymphocytes in addition to the remarkable elevation observed in CRP and IL-6 levels, which are inflammatory markers, in lymphocytes, which are significantly mitigated by concomitant administration of different Kn doses. The profound increase in hepatic TNF-α expression accompanied by CP injection was already detailed in previous studies [3,30].

CD95 (sometimes referred to as Fas) is a member of the tumor necrosis factor receptor (TNFR) superfamily [37]. Immature dendritic cells (DCs) undergo maturation upon CD95 activation, which increases the expression of MHC class II and DC-lysosome-associated membrane proteins (LAMPs) and triggers the release of pro-inflammatory cytokines, including IL-1b and TNF-α [38]. Moreover, CP induces plasmalemmal destabilization and increases membrane fluidity, which causes the death receptor CD95/Fas activation; such changes activate the extrinsic pathway of apoptosis and contribute to cell death [39]. Likewise, CP significantly elevated CD95 in isolated lymphocytes in our study, emphasizing its crucial role in both inflammation and apoptosis, and this observation is in concordance with previous reports [40,41]. Intriguingly, Kn attenuated CD95 levels remarkably at all doses, indicating its anti-inflammatory and anti-apoptotic properties.

Cisplatin intoxication shifts the balance between pro-and anti-apoptotic signals towards proapoptotic cascade [42]. TNF- is a protein that is involved in inflammatory responses and is intimately linked to apoptosis [43]. Since the interaction between oxidative stress and inflammation is a hallmark of cell death [44], oxidative stress caused by CP, which is believed to be via activation of the NF-ĸB pathway, resulted in inflammation and apoptosis [2]. In addition, when cells experience apoptosis, caspases 8 and 9 become activated, which are initiator caspases, resulting in activation of executioner caspases, including caspases 3 and 7 [45]. Furthermore, executioner caspase-3 activates cellular proteins and DNA fragmentation factors, resulting in apoptosis-mediated alterations [46]. AKT/PKB is a serine/threonine kinase that belongs to a protein family that also contains AKT1, AKT2 and AKT3. Cell proliferation, differentiation, apoptosis and cancer are all regulated by the AKT pathway [47,48]. Of note, it has been reported that the activation of AKT promotes cell survival, whereas inhibition of AKT activity promotes apoptosis in a variety of cancer cells [49].

Therefore, in the current work, it was predicted that hepatic oxidative stress brought on by a single dose of CP (7.0 mg/kg) would result in apoptosis, which was supported by hepatic AKT downregulation, pAKT/AKT ratio elevation and enhanced caspase-3 gene expression in rat liver tissue.

Our findings concur with those of Hassan et al., (2020) and Neamatallah et al., (2018), which demonstrate that CP treatment causes apoptosis in cells [2,3], as well as Dong Li et al., (2019), who reported that the level of p-Akt protein expression was also found to be significantly elevated when induced by oxidative stress in chondrocyte apoptosis [50].

Intriguingly, Kn, in the present study, decreased the CP-induced elevation in caspase-3 expression and significantly restored the pAKT/AKT ratio and AKT gene expression in a dose-dependent manner. Since increased ROS and pro-inflammatory cytokines have been documented to produce hepatocyte apoptosis and cell death [25,31,51,52], the decreased expression of caspase-3, pAKT/AKT ratio restoration and AKT gene upregulation in response to Kn treatment may be secondary to its antioxidant and anti-inflammatory activities. Our research clearly demonstrated that Kn provided protection against the liver and lymphocyte damage caused by cisplatin. We were able to directly attribute this cytokinin anti-apoptotic effect to AKT signaling, identifying it for the first time in the literature.

This strongly suggests that Kn might directly activate anti-apoptotic signaling at the cellular level, which is why we think it is significant.

We believe that the current research on receptor-mediated activity may play a significant additive role in elucidating the other robust tissue protection mechanisms of Kn that have been previously hypothesized or described.

## 5. Conclusions

In summary, Kn treatment mitigated the oxidative stress status and inflammation in the rats. Oxidative stress parameters as well as TNF-α in both livers and lymphocytes were strongly attenuated by Kn in this animal model of cisplatin-induced hepatotoxicity and lymphotoxicity. Moreover, the anti-apoptotic effects of Kn were accompanied by suppression of AKT and Caspase-3 expression in livers as well as CD95 in lymphocytes of cisplatin-treated rats. Our data suggest that Kn protects rat livers and lymphocytes against cisplatin-induced toxicity through antioxidant, anti-inflammatory and anti-apoptotic mechanisms. Therefore, we conclude that our results further imply that administration of Kn slows down the progression of hepatotoxicity as well as lymphotoxicity by inhibiting oxidative damage, inflammation and apoptosis.

## Figures and Tables

**Table 1:** List of primer sequences.

S. No	Gene	Sequences (5′-3′)	
		Forward	Reverse
1	AKT	GTC GCC TGC CCT TCT ACA AC	CAC ACG ATA CCG GCA AAG AA
2	Caspase-3	GTG GAA CTG ACG ATG ATA TGG C	CGC AAA GTG ACT GGA TGA ACC
3	GAPDH	ACC CAG AAG ACT GTG GAT GG	CAC ATT GGG GGT AGG AAC AC

## Data Availability

Not applicable.
